# More than meets the eye: The attentional blink in multisensory environments. Commentary on Kranczioch and Thorne

**DOI:** 10.2478/v10053-008-0141-x

**Published:** 2013-09-20

**Authors:** Till R. Schneider

**Affiliations:** Department of Neurophysiology and Pathophysiology, University Medical Center Hamburg-Eppendorf, Germany

**Keywords:** attentional blink, cross-modal, auditory-visual, reliability

## Abstract

Temporal fluctuations of attention can influence performance of cognitive tasks substantially. A common paradigm to investigate temporal fluctuations of attention is the attentional blink paradigm. Kranczioch and Thorne (2013) report new evidence for the impact of auditory stimuli on the visual attentional blink in the current issue of *Advances in Cognitive Psychology*.

## Commentary

Visual attention is not a stable state but rather a dynamical process, which changes from moment to moment depending on external events, task-demands, or internal states. In this issue of *Advances in Cognitive Psychology*, Kranczioch and Thorne (2013) demonstrate that visual perception can be enhanced by input from another sensory modality in an attentional blink (AB) paradigm. Preceding stimulation of another sensory modality can enhance visual processing - an effect called *cross-modal cueing* (Spence & McDonald, 2004). In cross-modal cueing paradigms, an auditory stimulus, for example, draws attention toward a visual stimulus. The authors investigated whether additional auditory input improved the target identification and whether the timing of the concurrent auditory stimulation had differential effects on the target performance.

The *AB* refers to a very brief reduction of attention that is triggered by two targets (T1 and T2) when presented in close temporal vicinity. An important characteristic of most AB experiments is that targets are presented in a rapid stream of visual distractor stimuli. Kranczioch and Thorne (2013) investigated whether concurrent auditory stimulation affected the visual AB and engaged classical split-half and test-retest measures to test the reliability of their findings. They report an improvement of T2 performance due to concurrent auditory-visual stimulation in the AB period in two cases: An auditory cue either precedes a visual target or is presented simultaneous with it. These findings are in contrast to results of a previous investigation by Olivers and Van der Burg (2008), showing that the AB largely disappeared with simultaneous but not with alerting auditory cues. According to Olivers and Van der Burg’s argumentation, this performance improvement is a perceptual effect, mediated mainly automatically, and cannot be explained by cross-modal attentional cueing. The findings of Kranczioch and Thorne, however, suggest that cross-modal cueing and strategic control may influence T2 performance as well. Such discrepant results raise new questions on the nature of cross-modal interactions affecting the AB. One reason for the observed discrepancy might be the validity of the auditory cue. In the experiment of Kranczioch and Thorne, the auditory cue always reliably predicted T2, whereas in the study by Olivers and Van der Burg, it did not. Slight differences between the two studies also exist in the exact timing of targets, distractors, and masks. Differences in stimulus onset between visual and auditory stimuli might well influence the effectiveness of cross-modal interactions. Therefore future studies should systematically investigate the influence of visual-auditory stimulus-onset asynchronies in order to better characterize the cross-modal attenuation of the AB.

The authors have to be complimented not only for their attempt to replicate previous results, but also for their effort to compute classical measures of reliability on the data. This is important for confidence in the present results and also beneficial for research on the AB in general. Split-half and test-retest reliability tests, however, revealed mixed results: Split-half reliability was found in the first of two sessions but not in the second. Test-retest reliability was observed in the unisensory AB, but not for the cross-modal conditions. These findings underline the sensitivity of the AB for various factors, which are also reported in the literature.

One further indication that the AB is susceptible to various influences is the common observation that it cannot be found in all subjects. Therefore many investigators split their sample of participants into “blinkers” and “non-blinkers.” This tendency suggests that temporary states (such as arousal or fatigue), enduring cognitive dispositions (such as distractibility), or disorders (such as attention deficit disorders) play a major role for the presence of the AB. Instead of splitting their participants into two groups, Kranczioch and Thorne (2013) identified dispositions which might be related to individual differences in attentional blinking. The authors investigated, for example, whether the distractibility of the participants - as measured with a German version of the Cognitive Failures Questionnaire (Broadbent, Cooper, FitzGerald, & Parkes, 1982; Lumb, 1995) - has an impact on the effect of cross-modal cueing. According to the authors’ reasoning, individuals might be at different degrees susceptible to auditory distractors, and thus would show differences in the size of the cross-modal cueing effect. Interestingly, individuals with high distractibility scores benefit less from the auditory cue when it was presented simultaneously to T2. There was no association between distractibility scores and performance when auditory cues were presented before T2. This implies that cognitive dispositions and traits have to be taken into consideration when interpreting AB results as also suggested by other researchers (e.g., Dale & Arnell, 2010).

The AB has been intensely investigated in the past two decades (Martens & Wyble, 2010). Several models have been proposed which are capable of explaining certain aspects of the AB, but none of the models is capable of explaining all aspects (Janson & Kranczioch, 2011). Frequently investigated factors influencing the AB are stimulus-onset asynchronies, type of T1 and T2 tasks, and visual properties of targets and distractors. The results are manifold as manipulations of one of these factors can increase or decrease the size of the AB. The AB seems to be a phenomenon that can be easily destroyed when one of these presentation parameters are slightly altered (Müsch, Engel, & Schneider, 2012). Thus, studies trying to replicate previous findings are of utmost importance.

The size of the AB can be effectively modulated by the salience of the target. Highly salient targets are capable of attracting attention and have been reported to reduce the size of the AB (Landau & Bentin, 2008; Müsch et al., 2012). Emotionally arousing targets, for example, have been reported to reduce the size of the AB, as they capture attention more easily (Anderson & Phelps, 2001; Keil, Ihssen, & Heim, 2006). The studies by Kranczioch and Thorne (2013) and by Olivers and Van der Burg (2008) are the first ones that demonstrate that performance in a visual AB task can be improved by a tone presented simultaneously with the targets.

On the neuronal level it has been suggested that pre-T1 neuronal activity is related to subsequent performance in T1 and T2 detection (for a review, see Janson & Kranczioch, 2011). Especially oscillatory activity in the beta-band (13-30 Hz) before T1 might be indicative of brain states that are beneficial for the task demands of the typical AB paradigm. In the case of cross-modal enhancement in the AB task, one can only speculate about the potential neuronal mechanisms. One possibility would be that oscillatory activity in the pre-T1 interval is reset due to the auditory stimulus onset and thereby modulating the processing of the visual targets. Phase-reset of ongoing oscillatory activity is discussed as one important mechanism of cross-modal interactions (Kayser, Petkov, & Logothetis, 2008; Lakatos, Chen, O’Connell, Mills, & Schroeder, 2007). This neuronal mechanism might also account for the cross-modal enhancement effect and could be investigated by systematically testing onset asynchronies between sensory inputs within this paradigm. In conclusion, Kranczioch and Thorne (2013) demonstrate that performance in the AB task can be effectively improved by cross-modal interactions and set the stage for further investigations on this effect and on the neuronal mechanisms which are mediating this effect.
